# Supplementary material for: Cannabidiol Selectively Binds to the Voltage-Gated Sodium Channel Nav1.4 in Its Slow-Inactivated State and Inhibits Sodium Current
Source: Biomedicines. 2021 Sep 2;9(9):1141. doi: 10.3390/biomedicines9091141 (PMC8465134; doi:10.3390/biomedicines9091141)
Supplement: Supplementary file 1 [file biomedicines-09-01141-s001.zip › biomedicines-1361327-supplementary.pdf]

Supplementary data

Cannabidiol inhibits sodium current via selectively binds to the voltage-gated sodium channel $Na_V1.4$ in its slow-inactivated state

Chiung-Wei Huang [1,2], Pi-Chen Lin [3], Jian-Lin Chen [4] and Ming-Jen Lee [5,*]

1 Department of Post Baccalaureate Medicine, Kaohsiung Medical University, Kaohsiung, 80708, Taiwan; g10054b@kimo.com
2 Department of Physiology, Kaohsiung Medical University, Kaohsiung, 80708, Taiwan
3 Department of Internal Medicine, Division of Endocrinology and Metabolism, Kaohsiung Medical University Hospital, Kaohsiung, 80708, Taiwan; pichli@kmu.edu.tw
4 Department of Physiology, National Taiwan University College of Medicine, Taipei, 100233, Taiwan; r07441009@g.ntu.edu.tw
5 Department of Neurology, National Taiwan University Hospital, Taipei, 100233, Taiwan
* Correspondence: mjlee@ntu.edu.tw; Tel.: +886-2-2312-3456 (ext. 65336)

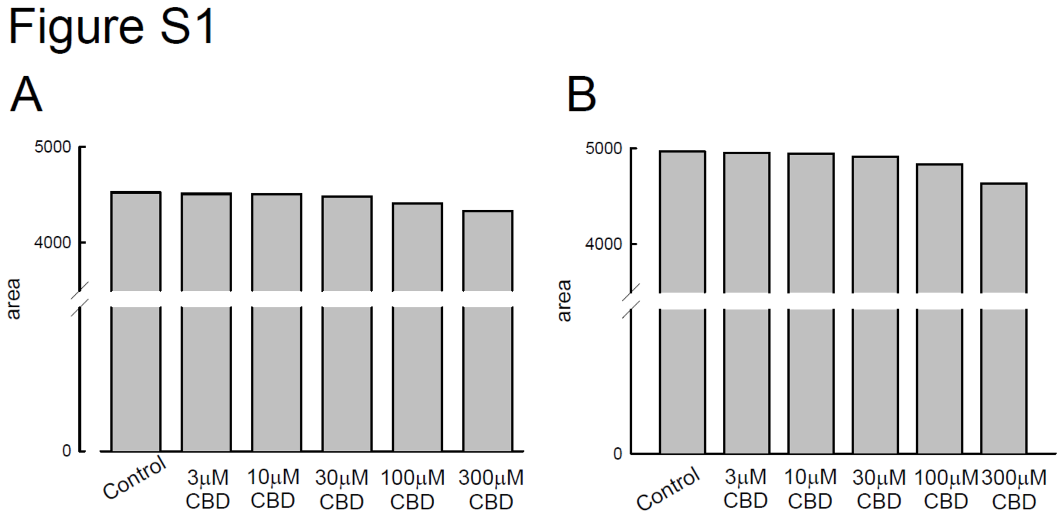

Figure S1. The area under the recovery time course curves obtained from depolarization at −10 and −80 mV

(A) Cumulative results from the area under the recovery time course curves obtained from the average data in Figure 5A.

(B) Cumulative results from the area under the recovery time course curves obtained from the average data in figure 5B.
